# Flexible Screen‐Printed Electrochemical Sensor for Alkaline Phosphatase Detection in Biofluids for Biomedical Applications

**DOI:** 10.1002/open.202500113

**Published:** 2025-04-13

**Authors:** Panagiota M. Kalligosfyri, Antonella Miglione, Alessia Esposito, Raghad Alhardan, Gabriella Iula, Iclal Atay, Ibrahim A. Darwish, Sevinc Kurbanoglu, Stefano Cinti

**Affiliations:** ^1^ Department of Pharmacy University of Naples Federico II 80131 Naples Italy; ^2^ Department of Analytical Chemistry Faculty of Pharmacy Ankara University 06560 Ankara Türkiye; ^3^ The Graduate School of Health Sciences Ankara University 06110 Ankara Türkiye; ^4^ Department of Pharmaceutical Chemistry College of Pharmacy King Saud University P.O. Box 2457 Riyadh 11451 Saudi Arabia; ^5^ Bioelectronics Task Force at University of Naples Federico II Via Cinthia 21 80126 Naples Italy; ^6^ Sbarro Institute for Cancer Research and Molecular Medicine Center for Biotechnology, College of Science and Technology Temple University Philadelphia, Pennsylvania 19122 U. S. A.

**Keywords:** alkaline phosphatase, biosensors, diagnostics, electroanalysis

## Abstract

Alkaline phosphatase (ALP) is an enzyme present in the human body responsible for the dephosphorylation of phosphorylated chemical species. It is primarily expressed in organs such as bones, liver, intestine, and placenta during pregnancy, playing a crucial role in cellular processes like gene expression, transport, and metabolism. Physiological ALP levels vary with age and sex, with normal serum ranges for healthy adults between 40 and 190 U/L. Alterations in ALP levels can be indicative of several pathologies, including cancer diagnosis and metastasis, as well as bone growth dysfunctions and hypophosphatasia. Conventional methods for ALP detection often require complex assay principles, extensive sample pretreatment, and trained personnel. Herein, the development of a portable, flexible electrochemical sensor fabricated through screen‐printing to monitor ALP levels in biological samples is introduced. The flexible electrochemical sensor, characterized by high efficiency, sustainability, low cost, and ease of disposal, achieves detection limit as low as 0.03 and 0.08 U/L, respectively, in buffer solution and human serum samples, and a satisfactory repeatability lower than 10%. This simple sensor configuration approach enables real‐time disease monitoring and improves access to point‐of‐care diagnostics, paving the way for affordable, decentralized sensors that support early diagnosis and better healthcare.

## Graphical abstract

A sustainable, flexible electrochemical sensor enables simple alkaline phosphatase detection in serum. Fabricated via screen‐printing, it offers a low‐cost, disposable, and eco‐friendly diagnostic solution. With a detection limit of 0.08 U/L and a repeatability below 10%, it supports real‐time, decentralized healthcare. These results validate the “less‐is‐more” concept, proving complex designs are not always necessary for optimal performance.

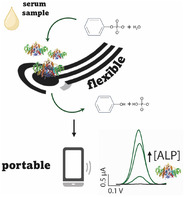

## 1 Introduction

Alkaline phosphatase (ALP) is a key enzyme in various biological processes, including metabolism, gene expression, and molecular transport.^[^ ^1^ ^]^ As an emerging biomarker in diagnostics, ALP levels in serum samples provide valuable insights into disease states and treatment efficacy. In adult humans, serum ALP levels typically range from 40 to 190 U/L, while in pregnant women and children, they can exceed 500 U/L.^[^ ^1^ ^]^ Elevated ALP levels might be associated with several pathological conditions, such as liver dysfunction, hepatitis, stroke,^[^ ^2^ ^]^ and prostate cancer,^[^ ^3^ ^]^ highlighting its importance in clinical assessments. Meanwhile, abnormally low ALP levels are linked to nutritional deficiencies (e.g., zinc and magnesium), hypothyroidism,^[^ ^4^ ^]^ hypophosphatasia, and Wilson's disease, which impact both bone and liver function.^[^ ^5^ ^]^ Malnutrition and certain medications may also contribute to decreased ALP levels.^[^ ^6^ ^]^

Traditional ALP detection methods include colorimetric and fluorescence methods, which have been widely used due to their simplicity and sensitivity.^[^ ^7^ ^]^ Colorimetric and fluorescence methods detect ALP by substrate dephosphorylation, producing color changes or light emission, often requiring laboratory‐based instrumentation. Additional techniques, including histochemistry, northern and western blots, and enzyme‐linked immunosorbent assays (ELISA) with colorimetric detection, also enable ALP detection in various applications.^[^ ^7^, ^8^, ^9^, ^10^ ^]^ Moreover, among all the existing approaches, the Scharer rapid phosphatase colorimetric test is commonly employed for food safety testing.^[^ ^11^ ^]^ However, while these methods offer high sensitivity, they come with several drawbacks. Colorimetric and fluorescence techniques often require complex instrumentation reducing their portability and practicality for on‐site use. Additionally, these methods can be time‐consuming and demand substantial hands‐on effort, making them less suitable for rapid or field‐based applications. The need for faster detection assays and portable devices has driven the development of biosensing technologies, offering more efficient, real‐time, and user‐friendly solutions for ALP detection.^[^ ^12^ ^]^

Electrochemical sensors play a significant role in ALP detection with several strategies reported to enhance their performance.^[^ ^13^ ^]^ These include surface modification of working electrodes with nanocomposite mixtures from bioactive glass and multiwalled carbon nanotubes, gold nanoparticles, quantum dots, nanostructured metal oxides, labeled probes, polypyrrole films, enzymes, antibodies, and carboxyl groups for further biomolecule immobilization.^[^ ^14^, ^15^, ^16^, ^17^, ^18^ ^]^ These approaches can also be combined and aim to improve electrode conductivity, sensitivity, and affinity for specific target biomarkers.^[^ ^12^ ^]^ Given that ALP levels as low as 20 U/L are considered deficient^[^ ^2^, ^19^ ^]^ and based on reported sensors for ALP detection,^[^ ^12^, ^20^, ^21^ ^]^ it appears that achieving the lowest detection limit utilizing overly complex strategies may not be the ultimate goal for point‐of‐care applications. Instead, focusing should be on maintaining sensitivity within the clinically relevant range, ensuring the sensor remains practical and effective for clinical applications while utilizing simpler protocols and cost‐effective materials.

In this context, the present work introduces a simple yet effective electrochemical sensor. Flexible polyester sheets served as the biosensor substrate, while conductive inks were screen‐printed to create the three‐electrode system. ALP detection was achieved by introducing its specific substrate, namely, disodium phenyl phosphate. The sensor was analytically characterized, demonstrating a linear range from 15 to 500 U/L with a low limit of detection (LOD) down to 0.03 U/L in buffer. A matrix effect study enabled the quantification of ALP in spiked, commercially available serum samples diluted to 20%, achieving a LOD of 0.08 U/L, confirming the potential of the developed sensor for clinical diagnostics.

## 2 Results and Discussion

### 2.1 Assay Principle of the Sensor

**Figure 1:**
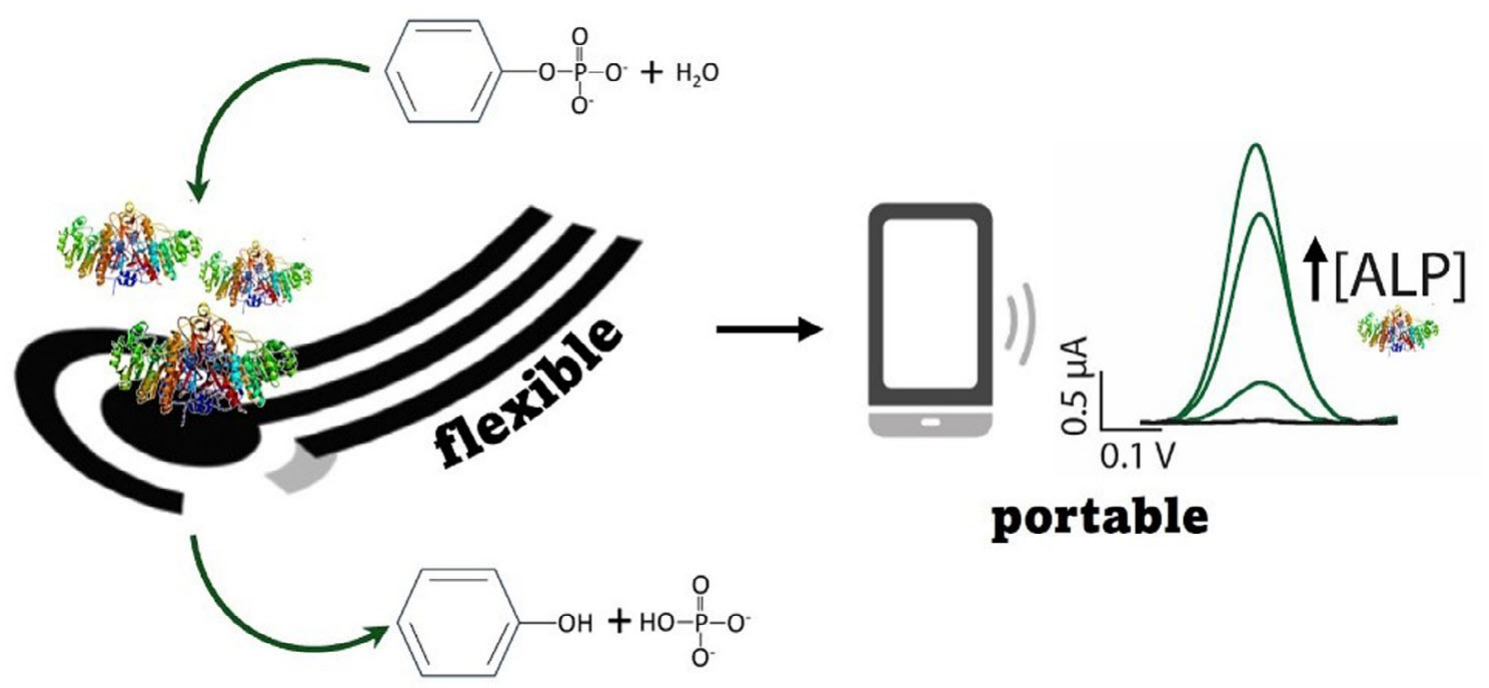
Assay principle of the flexible and portable SPE system.

Electrochemical sensors for detecting ALP typically exploit the enzyme's ability to convert a substrate into an electrochemically active product that can be measured. Common substrates used for this purpose include disodium phenyl phosphate, hydroquinone diphosphate, and ferrocene‐based compounds. Among these, disodium phenyl phosphate is frequently chosen.^[^ ^12^ ^]^ In this reaction, ALP hydrolyzes disodium phenyl phosphate, cleaving the phosphate group and producing phenol, the electroactive species detected by the sensor. In the present study, the ALP‐containing sample is applied to a screen‐printed working electrode on a flexible, cost‐effective, and portable polyester substrate (**Figure** **1**).

The phenol generated undergoes electrochemical oxidation, producing a measurable current signal. Techniques such as cyclic voltammetry and differential pulsed voltammetry are employed to quantify the phenol concentration, which directly correlates with ALP activity in the sample. The greater the ALP activity, the more phenol is produced, leading to a stronger electrochemical signal.

### 2.2 Optimization Studies

**Figure 2:**
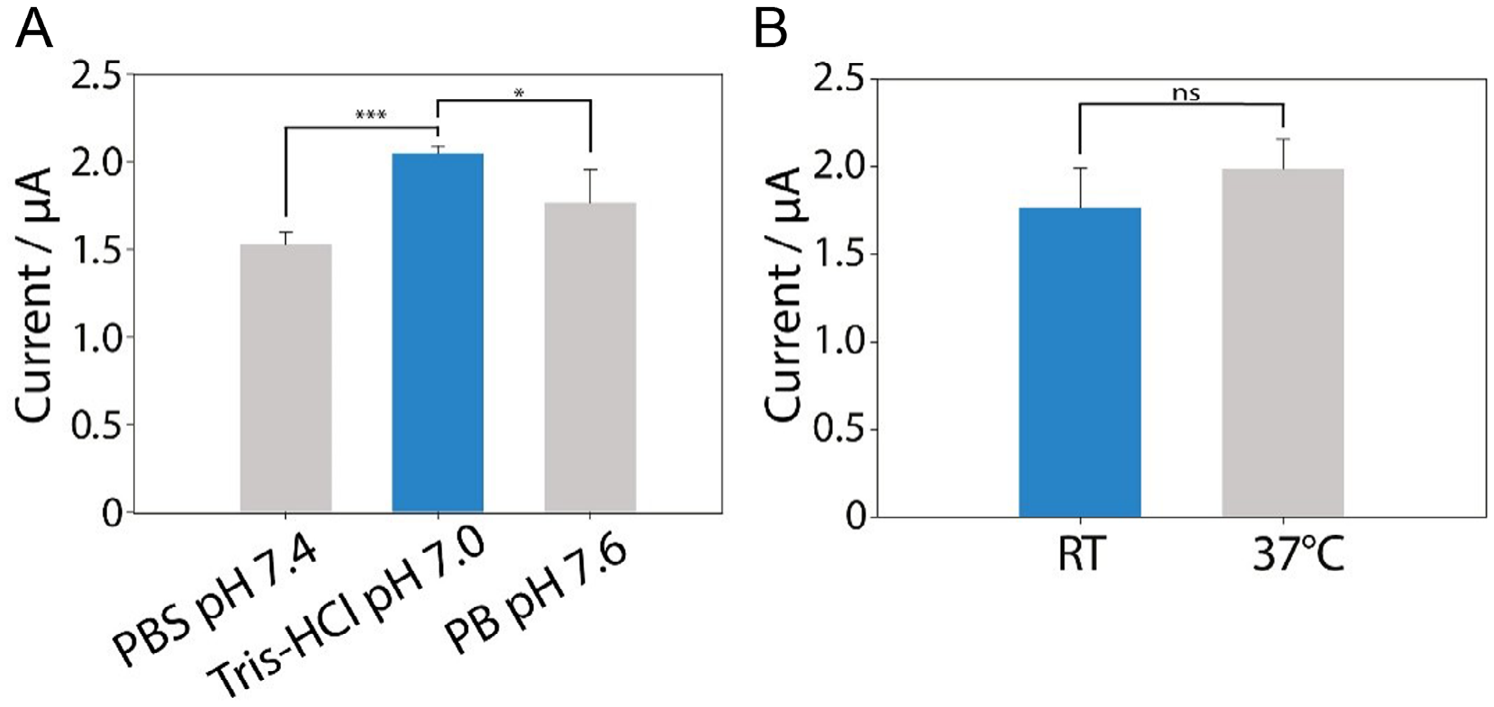
Investigation of A) the pH and buffer, and B) temperature on ALP enzyme performance. 200 U/L of ALP were tested in the presence of 5 mM disodium phenyl phosphate substrate. All the experiments were performed in triplicates in CV. CV parameters: initial potential of −0.1 V, vertex potentials of −0.1 and 0.9 V, step potential of 0.01 V, scan rate of 0.05 V s^−1^. Horizontal lines indicate the statistical significance between the groups: *** p < 0.001, ** p < 0.01, * p < 0.05, not significant (ns) p > 0.05.

To determine the optimal experimental conditions for the flexible screen‐printed electrodes (SPEs), optimization studies were conducted, assessing key experimental factors influencing enzyme's activity and biosensor performance. Various buffers with pH values ranging from 7.0 to 7.6, similar to those found in biological samples^[^ ^22^ ^]^ (e.g., serum), were tested. Among the buffers evaluated, Tris‐HCl buffer provided the highest and most reproducible biosensor response. Statistical analysis via *t*‐tests revealed that the reaction in Tris‐HCl buffer showed a significant difference compared to both phosphate buffer saline and phosphate buffer (**Figure** **2**A). This indicates that the use of Tris‐HCl buffer significantly enhanced sensor performance, making it the optimal choice for subsequent experiments.

As the goal of the proposed study was the development of a point‐of‐care device, we also evaluated the effect of temperature on biosensor's performance. Since the optimal enzymatic reaction temperature is 37 °C, with 25 °C showing up to 80% of the enzymatic activity,^[^ ^23^, ^24^ ^]^ we investigated the sensor's performance at room temperature (RT) to explore a more equipment‐free approach. Statistical *t*‐tests were performed via Python, and the results showed no statistically significant difference (p = 0.07, >0.05) between the signal responses at RT and 37 °C (Figure 2B). This indicates that temperature does not have a strong effect on the signal response. Therefore, we proceeded with the optimization and development of the biosensor at RT to enhance practicality and accessibility, without compromising its performance.

**Figure 3:**
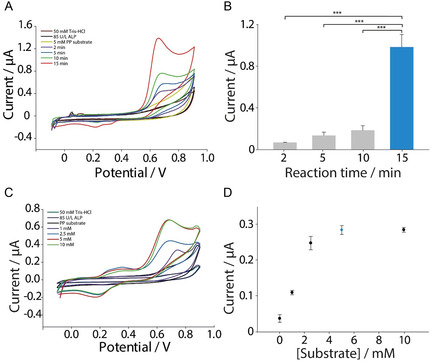
A) Optimization of the reaction time, i.e., the incubation time of 85 U/L of ALP enzyme in presence of 5 mM of disodium phenyl phosphate (PP) substrate in 50 mM of Tris‐HCl buffer, pH 7.00. B) Histograms represent the increase of the reaction signal over incubation times ranging from 2 to 15 min incubation time. Statistical analysis was performed using paired *t*‐test. C) Investigation of the optimal PP substrate concentration using 85 U/L of ALP enzyme with a 15‐min incubation time in 50 mM of Tris‐HCl buffer, pH 7.00. D) Biosensor response as a function of increasing substrate concentration. All the experiments were performed in triplicates in CV. CV parameters: initial potential of −0.1 V, vertex potentials of −0.1 V and 0.9 V, step potential of 0.01 V, scan rate of 0.05 V s^−1^. *** p < 0.001, ** p < 0.01, * p < 0.05.

Various reaction times ranging from 2 to 15 min were tested using 85 U/L of ALP enzyme and 5 mM of the specific substrate. A characteristic peak was increased by increasing incubation time at 0.65 V in the CV measurements. The highest current response was observed at a 15‐min incubation time, which was therefore selected as the optimal reaction duration (**Figure** **3**A).

A paired *t*‐test was conducted to compare the 2‐, 5‐, and 10‐min incubation times with the 15‐min interval. The statistical analysis was performed using a Python script to conduct paired *t*‐tests for each time interval against the 15‐min reaction time. The results indicate that the 15‐min incubation time provided the highest current response, with no statistically significant differences among the other time intervals (Figure 3B). Based on these findings, the 15‐min incubation time was selected for further studies, as it ensures optimal performance while minimizing analysis time. Therefore, no additional time intervals were explored, making this condition particularly suitable for point‐of‐care applications and on‐field testing.

After selecting the optimal incubation time, the substrate concentration was further investigated. Current responses were recorded for increasing concentrations of the specific substrate and compared to a blank sample without substrate addition. The signal increased with higher substrate concentrations until reaching a plateau at 10 mM (Figure 3C), as the enzyme was unable to further dephosphorylate the substrate. The study was conducted using 85 U/L of ALP with a 15‐min incubation time. The investigation of substrate concentration revealed a typical Michaelis–Menten behavior,^[^ ^25^ ^]^ as expected. As the substrate concentration increased, the reaction rate also increased, eventually reaching a plateau in the biosensor's response. This saturation point indicates that further increases in substrate concentration did not lead to a significant increase in the reaction rate. Based on these findings, a final concentration of 5 mM was selected as the optimal substrate concentration, as shown in Figure 3D.

### 2.3 Analytical Characterization of the Flexible Screen‐Printed Electrochemical System

The analytical performance of the optimized method was evaluated using increasing concentrations of ALP, prepared in 50 mM Tris‐HCl at pH 7.00. To enhance sensitivity, calibration curve measurements were performed using differential pulse voltammetry (DPV), which offers greater sensitivity compared to cyclic voltammetry (CV).^[^ ^26^ ^]^ Measurements were conducted in triplicate after a reaction time of 15 min in the presence of 5 mM of the specific substrate, as determined through optimization.

**Figure 4:**
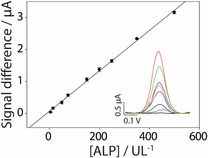
The calibration curve was obtained in a buffer solution of 50 mM Tris‐HCl at pH 7.0, with increasing target concentrations ranging from 15 to 500 U/L. The inset displays the DPV curves. DPV parameters: E_start_: 0.2 V, E_end_: 0.8 V, E_step_: 0.01 V, E_pulse_: 0.1 V, t_pulse_: 0.02 s, scan rate: 0.02 V s^−1^. All the experiments were performed in triplicate.

The resulting calibration curve is described by the Equation y = 6.33x + 0.06, with an R^2^ = 0.99, where y‐axis values represent the change in signal intensity measured between the solution containing only the substrate at a 5 mM concentration, i.e., the blank sample, and the solution where the enzyme is present, leading to substrate dephosphorylation and subsequent oxidation. The *x*‐axis values correspond to the ALP concentration, expressed in U/L (**Figure** **4**).

The LOD was calculated as the ratio of three times the standard deviation of the blank to the slope of the calibration curve, resulting in a value of 0.03 U/L, demonstrating the assay's ability to detect even highly diluted samples without requiring additional pretreatment to reduce matrix effects. The repeatability of the developed assay was assessed in terms of the relative standard deviation (RSD%). The RSD% was calculated by dividing the standard deviation by the mean value of the measurements and multiplying the result by 100. This calculation demonstrates the repeatability of the assay, which in this case yielded an RSD% up to 8.5% for all calibrators in solution.

### 2.4 Matrix Effect Investigation

**Figure 5:**
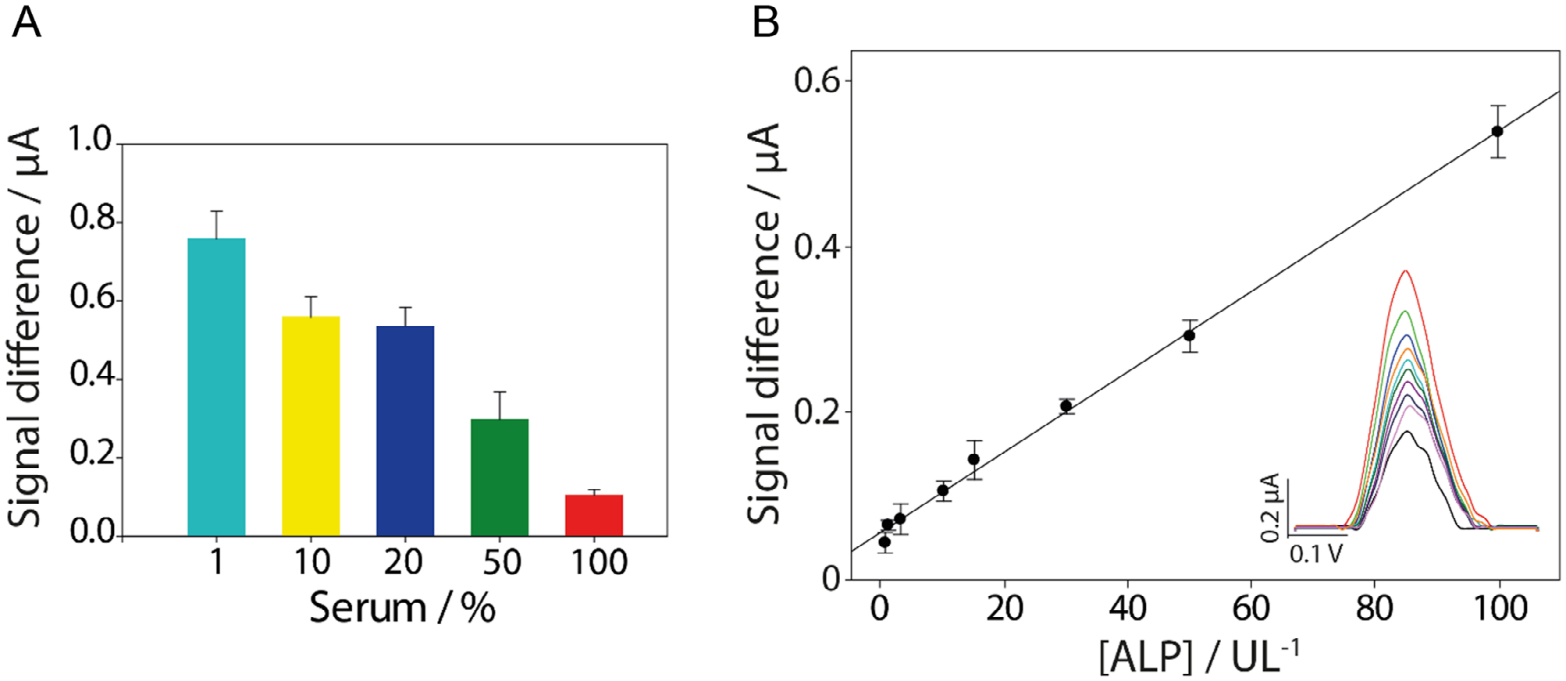
A) Study of matrix effect utilizing different serum dilutions in the presence of 250 U/L of ALP and a substrate concentration of 5 mM. 100% of serum corresponds to undiluted serum sample. The results are presented as the signal (current) difference relative to the reference blank. B) Calibration curve performed in 20% serum with increasing ALP concentrations ranging from 0.5 to 100 U/L. The inset displays the DPV curves. DPV parameters: E_start_: 0.2 V, E_end_: 0.8 V, E_step_: 0.01 V, E_pulse_: 0.1 V, t_pulse_: 0.02 s, scan rate: 0.02 V s^−1^. All the experiments were performed in triplicate.

Following the analytical performance evaluation of the biosensor, attention was focused on assessing ALP behavior and the sensor's detection capability in a system that mimics the biological environment. In undiluted serum samples, various chemical species and biomolecules are present, potentially interfering with measurements and altering the detected signal. This phenomenon, known as the matrix effect, can lead to a reduction in the observed current response. To mitigate this effect, different serum dilutions, namely, 1, 10, 20, and 50%, were prepared using 50 mM Tris‐HCl buffer at pH 7.0 and systematically evaluated to determine the optimal conditions for accurate ALP detection (**Figure** **5**A).

Despite the histogram clearly showing that the current signal difference is higher at a serum dilution of 1%, as expected due to the lower matrix effect, this dilution was not chosen as the optimal condition for subsequent experiments. The increase in current observed at this dilution was not significant enough to justify such a high dilution of the sample. Additionally, a 20% serum dilution was selected to avoid excessive sample dilution while staying within the clinically significant range. After evaluating these considerations and examining the system's behavior at other dilution levels, a 20% serum dilution was chosen as a balanced compromise between sensitivity and maintaining a sufficiently high current signal.

### 2.5 Analytical Characterization in Serum Samples

Commercial human serum (Sigma‐Aldrich, St. Louis, MO, USA) was used in this experimental section. The analytical performance of the system was further evaluated in 20% serum at increasing concentrations of ALP, ranging from 0.5 to 100 U/L. After adding the substrate, DPV was utilized to record the current responses of the sensor, as described previously. A linear range was obtained (Figure 5B) following a linear regression fitting described by the Equation y = 0.44x + 0.005 with R^2^ = 0.98, where x represents the amount of spiked ALP in U/L in the 20% diluted serum samples, and y represents the current signal change between the blank reference and the measurement of the solution where dephosphorylation occurs. Finally, the LOD was found to be 0.08 U/L in the fivefold diluted samples, which corresponds to 0.4 U/L in the undiluted samples, considering the dilution factor. This demonstrates the potential of the proposed flexible biosensor for real clinical applications. The %RSD was also calculated for all spiked serum samples and was up to 9%, ensuring the sensor's repeatability.

Despite consisting of a homemade SPE without any surface modification, our biosensor achieved a LOD comparable to previously reported electrochemical biosensors for alkaline phosphatase detection^[^ ^18^, ^27^, ^28^, ^29^, ^30^, ^31^, ^32^ ^]^ (**Table** **1**). Notably, our approach does not rely on complex labeling strategies, nanomaterial modifications, incubators, or lengthy preparation protocols, making it a simpler and more cost‐effective alternative for ALP detection. The ability to reach a clinically significant LOD using an unmodified electrode highlights the potential of our sensor for rapid and accessible diagnostics, particularly in point‐of‐care applications.

**Table 1:** Comparison of electrochemical methods for ALP detection using different electrode types, modification strategies, and detection methods.

Electrode typea)	Modification	Detection method	Sample	Reaction temperature	Assay time [min]	LOD (U/L)	Ref.
Screen‐printed	N/A	DPV	Buffer	37 °C	27	0.4	[27]
Glassy carbon electrode	N/A	SWV	Buffer	37 °C	10	3	[28]
Fluorine‐doped Tin Oxide electrodes	Gold nanoclusters‐decorated Ag@SiO2	Photoelectrochemical	Buffer	NS	40	0.022	[29]
Commercial screen‐printed	Au‐nanodendroids, and graphene oxide nanocomposite	EIS	Serum	NS	30	9.10	[30]
Au‐electrodes	ssDNA probe	DPV	Buffer	RT	60	1.48	[31]
Au‐electrodes	Sulfur groups	SWV	Buffer	37 °C	60	1.64	[32]
Glassy carbon electrode	Bioactive glass, multiwalled carbon nanotubes, and PVA	EIS	Spiked serum	NS	NS	2.07	[18]
Screen‐printed	N/A	DPV	Spiked serum	RT	15	0.4	This work

a) N/A: not applicable; PVA: polyvinyl alcohol; EIS: electrochemical impedance spectroscopy; Au: gold; ss: single stranded; NS: not specified; RT: Room Temperature; SWV: square wave voltammetry.

## 3 Conclusion

In the present study, we adopted a minimalistic approach, embracing the principle of “less is more” to develop a simple yet effective enzymatic assay using flexible SPEs. This work introduces a novel flexible electrode sensor for detecting alkaline phosphatase in serum, designed with a focus on simplicity and efficiency. Traditional sensor development often leans toward complexity, under the assumption that more sophisticated systems lead to greater accuracy. However, our approach challenges this notion, demonstrating that simpler methods can yield equally effective results. The sensor features a streamlined design that requires minimal sample preparation and pretreatment, i.e., dilution step, relying on direct electrochemical measurement through a portable potentiostat connected to a smartphone or a portable computer. This stands in contrast to more complex techniques that involve multiple steps and extensive data processing. Despite its simplicity, the sensor shows sensitivity and accuracy comparable to more complicated systems.^[^ ^12^, ^21^ ^]^ Its clinically significant LOD aligns with the lower end of the normal ALP range in human serum (20–40 U/L) and can detect ALP levels as low as 0.4 U/L (0.08 U/L in 20% serum samples), making it suitable for identifying ALP deficiency. These results validate the “less‐is‐more” concept, proving that complex designs and procedures are not always necessary for optimal performance. The study highlights the potential of this user‐friendly, portable sensor for clinical practice, offering a complementary point‐of‐care diagnostic tool that can assist healthcare providers in making informed decisions for better disease management and treatment outcomes.

## 4 Experimental Section

4.1

4.1.1

##### Flexible SPE Preparation

The flexible SPEs were fabricated in‐house, as previously reported.^[^ ^33^, ^34^ ^]^ The flexibility of the electrodes was attributed to the use of a flexible polyester substrate (Autostat HT5, 125 μm, MacDermid, UK) as the printing platform. Carbon ink (Sunchemical, Parsippany, NJ, USA) was used for the printing of the working and counter electrodes, while conductive silver/silver chloride ink (Loctite, Henkel Italia Srl, Italy) was used for the reference electrode. The SPEs were treated at 100 °C for 30 min to dry and stabilize the ink. Prior to each measurement, adhesive tape was applied to the electrochemical sensor to prevent spreading and leakage of the sample, ensuring proper functioning of the electrical components and creating a hydrophobic boundary that defined the sample deposition area.

##### ALP Detection

The proposed screen‐printed electrochemical sensor was applied for the detection of ALP by simply using the specific ALP substrate, namely, disodium phenyl phosphate, exploiting the flexibility and cost‐effectiveness of the sensor. Standard solutions were prepared in 50 mM Tris‐HCl buffer or commercially diluted serum samples in 50 mM Tris‐HCl. Then, ALP samples and the specific substrate were mixed to a final volume of 100 μL, achieving a final substrate concentration of 5 mM. The mixture, containing the enzymatic reaction, was incubated for 15 min at RT to allow the formation of enzymatic products. The signal responses were then recorded using the portable Sensitsmart potentiostat (PalmSens, Houten, The Netherlands) through DPV^[^ ^35^, ^36^ ^]^ technique with the following parameters: E_start_: 0.2 V, E_end_: 0.8 V, E_step_: 0.01 V, E_pulse_: 0.1 V, t_pulse_: 0.02 s, scan rate: 0.02 V s^−1^. The results were interpreted as the signal difference between the reference blank sample (current intensity obtained in the absence of target ALP) and the current intensity obtained in the presence of ALP. The ALP enzyme, the specific substrate, and the other common reagents were purchased by Sigma‐Aldrich (St. Louis, MO, USA).

## Conflict of Interest

The authors declare no conflict of Interest.

## Data Availability

The data that support the findings of this study are available from the corresponding author upon reasonable request.
